# Prognostic Significance of *in situ* Phenotypic Marker Expression in Diffuse Large B-cell Lymphomas

**Published:** 2007-11-11

**Authors:** Alexandar Tzankov, Philip Went, Stephan Dirnhofer

**Affiliations:** Department of Pathology, University Hospital Basel, Switzerland

**Keywords:** Diffuse large B-cell lymphoma, Phenotype, Prognosis, Tissue microarray, Receiver operating characteristic (ROC) curves

## Abstract

Diffuse large B-cell lymphomas (DLBCL) are the most common lymphoid malignancies, and encompass all malignant lymphomas characterized by large neoplastic cells and B-cell derivation. In the last decade, DLBCL has been subjected to intense clinical, phenotypic and molecular studies, and were found to represent a heterogeneous group of tumors. These studies suggested new disease subtypes and variants with distinct clinical characteristics, morphologies, immunophenotypes, genotypes or gene expression profiles, associated with distinct prognoses or unique sensitivities to particular therapy regimens. Unfortunately, the reliability and reproducibility of the molecular results remains unclear due to contradictory reports in the literature resulting from small sample sizes, referral and selection biases, and variable methodologies and cut-off levels used to determine positivity. Here, we review phenotypic studies on the prognostic significance of protein expression profiles in DLBCL and reconsider our own retrospective data on 301 primary DLBCL cases obtained on a previously validated tissue microarray in light of powerful statistical methods of determining optimal cut-off values of phenotypic factors for prediction of outcome.

## Introduction

Diffuse large B-cell lymphomas (DLBCL) are the most common lymphoid malignancies, comprising 35% to 40% of all adult non-Hodgkin lymphomas. This category encompasses all malignant lymphomas characterized by large neoplastic cells and B-cell derivation (Gatter and Warnke, 2001; Lossos, 2005; Mitterlechner et al. 2006). DLBCL may develop *de novo* (primary DLBCL) or arise from a previously indolent lymphoma (secondary, transformed DLBCL) at virtually every nodal or extranodal location. It is most commonly observed in cervical, axillar and mediastinal nodes, the stomach and the ileo-coecal region (Gatter and Warnke, 2001). DLBCL are accompanied by an aggressive clinical presentation with the need for highly effective chemotherapy regimens (e.g. Coiffier, 2005). Only about 60% of patients can be cured by rituximab, cyclophosphamide, doxorubicin, vincristine, prednisone (R-CHOP) and equivalent treatment regimens (Coiffier, 2005; Mitterlchner et al. 2006). The gold standard of predicting survival and stratifying patients for risk-adjusted therapy is the international prognostic index (IPI) (Shipp et al. 1993), which consists of easily assessable clinical and laboratory parameters: age, serum lactate dehydrogenase (LDH), stage, performance status and >1 extranodal sites involved. No such histopathologically-defined parameters exist and although the current World Health Organization (WHO) classification (Gatter and Warnke, 2001) accepts different morphologic variants and subtypes of DLBCL, their prognostic utility is hampered by a high rate of interobserver variation, which generally minimizes their influence in therapy. In the last decade, extensive studies of the clinical, phenotypic and molecular aspects of DLBCL have identified them as a heterogeneous group of tumors. These studies suggested new disease subtypes and variants with distinct clinical characteristics, morphology, immunophenotypes, genotypes or gene expression profiles associated with distinct prognoses or unique sensitivities to specific therapy regimens (Pileri et al. 2002; de Leval and Harris, 2003; Wright et al. 2003; Rosenwald and Staudt, 2003; Lossos, 2005; Morgensztern and Lossos, 2005; de Paepe and de Wolf-Peeters, 2006; Muris et al. 2006a). Unfortunately, the reliability and reproducibility of the molecular results remains unclear, and consequently translation into generally accepted standards to predict survival and stratify patients for risk-adjusted therapy has not taken place (for critical remarks see e.g. Hsi, 2001; de Leval and Harris, 2003; Gascoyne, 2004). Technical issues (antibody affinity), lack of standardization of evaluation procedures (definition of cut-off values) and poor study designs (small sample size and collection bias) are the most important factors hindering the efficient clinical translation of these molecular data. From the histopathological standpoint, some of these problems might be resolved by (A) application of monoclonal antibodies and good working fluorescent *in situ* hybridization (FISH) probes, (B) standardized high throughput analysis methods such as tissue microarrays (TMA) (Tzankov et al. 2005a), (C) powerful statistical methods and (D) consideration of both biological (tumor-specific) and clinical (patient-specific) parameters on thoroughly characterized study collectives.

Here, we review phenotypic studies on the prognostic significance of protein expression profiles in DLBCL. Furthermore, we reconsidered our own retrospective data on 301 primary DLBCL cases obtained on a previously validated TMA (Tzankov et al. 2003a; Went et al. 2004; Zinzani et al. 2005; Tzankov et al. 2006) in light of powerful statistical methods that determine the optimal cutoff values of phenotypic factors for efficient outcome prediction. Since DLBCL with plasma-blastic differentiation and acquired immunodeficiency syndrome-related lymphomas, as well as primary mediastinal B-cell lymphomas, are beyond the scope of our review, we refer to recent overviews on these rare variants (Carbone and Gloghini, 2005; Teruya-Feldstein, 2005; Levine, 2006; Savage, 2006).

## DLBCL Immunophenotype

DLBCL are derived from germinal center-(GC) or post-GC B-cells, and probably from extrafol-licularly-activated B-cells (Alizadeh et al. 2000; Gatter and Warnke, 2001; Pileri et al. 2002; de Leval and Harris, 2003; Rosenwald and Staudt, 2003; Lossos, 2005). The neoplastic cells usually express a series of antigens encountered on mature B-cells. Classical DLBCL are often CD19^+^, CD20^+^, CD79a^+^, BSAP^+^ (Gatter and Warnke, 2001; Torlakovich et al. 2002; Pileri et al. 2002; de Leval and Harris, 2003). The leukocyte common antigen (CD45) is absent in about 30% of immunoblastic and anaplastic DLBCL (Falini et al. 1990; Gatter and Warnke, 2001). Some immunoblastic DLBCL, particularly those derived from preterminally-differentiated post-CG B-cells (plasmablastic-, primary effusion- and ALK^+^ DLBCL), often do not express CD20, CD79a and BSAP, but express MUM1, VS38c, CD138, or immunoglobulin (Ig) heavy or light chains (Delecluse et al. 1997; Delsol et al. 1997; Gatter and Warnke, 2001; Carbone and Gloghini, 2005; Teruya-Feldstein, 2005; Savage, 2006). Lineage specificity in such cases therefore requires immunohistochemical analysis utilizing a broader marker panel. Importantly, considering B-lineage markers, the rituximab era highlights the importance of assessing CD20-status in DLBCL at primary diagnosis and at every sequential biopsy, since therapeutic efficacy is related to CD20 expression, which exposure to rituximab can abrogate (Davis et al. 1999; Held et al. 2006).

## Prognostic Significance of Immunophenotypic Cellular Differentiation Markers (Table 1)

**Figure 1:**
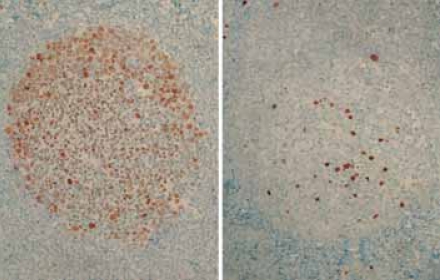
Reciprocal expression of Bcl-6 (left) and MUM1 (right) in normal germinal centers. Note striking Bcl-6 nuclear positivity in the centroblast-rich follicle dark zone and the majority of cells in the centrocyte-rich pale zone on the left as well as isolated MUM1^+^ cells within the germinal center on the right.

**Figure 2:**
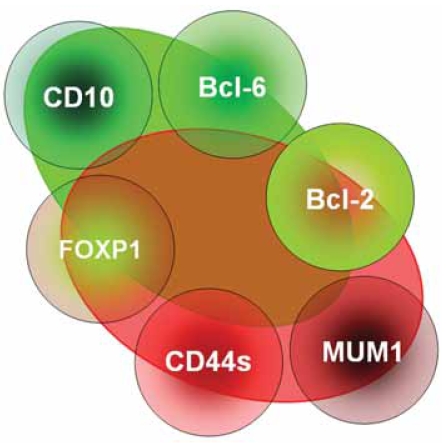
Cluster center analysis in our own series of diffuse large B-cell lymphomas considering expression of differentiation markers. The overlap of the red and green ellipsoids with the protein expression circles indicates cluster center tendency. Note the strict segregation of CD10- and MUM1 expression, but the comparatively low segregation of Bcl-6- and CD44s- and even lower Bcl-2- and FOXP1 expression within the diffuse large B-cell lymphoma clusters.

The concept of varying DLBCL histogenesis from GC and non-GC B-cells takes, similar to the Kiel and WHO lymphoma-classification concept, normal B-cell differentiation into consideration, a concept supported by gene expression profiling data (Alizadeh et al. 2000; Pileri et al. 2002; de Leval and Harris, 2003; Rosenwald and Staudt, 2003; Lossos, 2005). Non-neoplastic GC B-cells have a distinct protein expression profile (bcl-6^+^, CD10^+^, MUM1^−^ and CD44s^weakly+^). The expression of *BCL6* and *MUM1*, both involved in transcription regulation of genes important for lymphocyte activation and cell cycle control, is virtually reciprocal in normal B-cells (Fig. 1) (Shaffer et al. 2000; Falini et al. 2000). Protein expression in DLCBL in comparison to non-neoplastic B-cells is more complex (Fig. 2), suggesting deregulation of their gene expression, e.g. *MUM1* and *bcl-6* expression is not exclusive in DLBCL (Falini et al. 2000; Falini and Mason, 2002; Pileri et al. 2002; de Leval and Harris, 2003; Hans et al. 2004). Expression of each of the mentioned differentiation antigens has been found to be of prognostic significance in DLBCL, but these results remain somewhat controversial.

**Figure 3:**
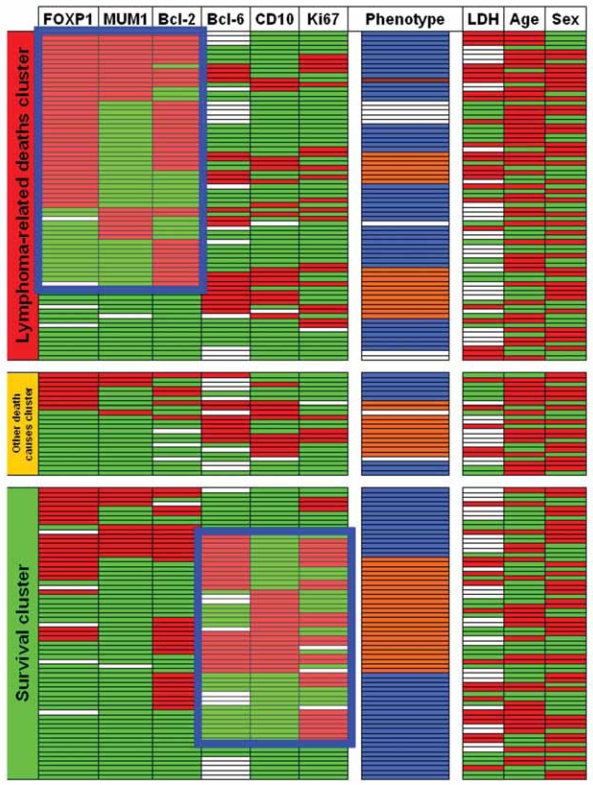
Outcome cluster analysis of phenotypic markers and phenotypes according to the “Hans’ algorithm” (Hans et al. 2004) and cut-off values from Table 2, as well as clinical parameters in diffuse large B-cell lymphomas (DLBCL). Cases expressing markers higher than the cut-offs, males, and patients >66 years are indicated in red, while negative cases, females, and individuals <66 are in green. Phenotypic germinal center DLBCL are indicated in orange, while non-germinal center DLBCL are in blue; only one DLBCL case (brown) co-expressed all three CD10, Bcl-6 and MUM1. Note the aggregation of FOXP1^+^, MUM1^+^ and Bcl-2^+^ cases in the lymphoma-related deaths cluster as well as the slight predominance of CD10^+^, Bcl-6^+^ and highly proliferative tumors in the survival cluster. Empty balks represent analysis failure or lacking LDH data.

**Figure 4:**
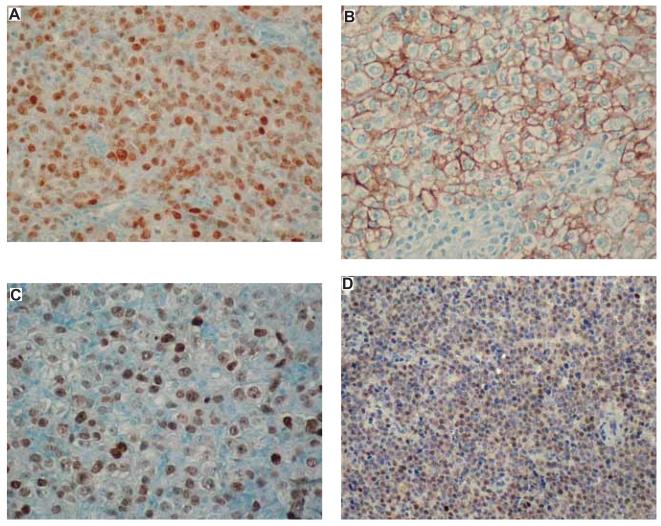
**A.** Expression of Bcl-6 in diffuse large B-cell lymphoma (DLBCL). Note intense and moderate nuclear signals in lymphoma cells as well as internal negative controls (endothelial nuclei). **B.** Expression of CD10 in DLBCL. Note intense membranous signals in lymphoma cells as well as internal negative controls (small lymphocytes). **C.** Expression of MUM1 in DLBCL. Note intense and moderate nuclear signals in lymphoma cells. **D.** Expression of cyclin E in diffuse large B-cell lymphoma. Note moderate and isolated intense nuclear signals in lymphoma cells.

Bcl-6 is a zinc finger sequence-specific transcriptional repressor specifically expressed on GC B-cells (Cattoretti et al. 1995; Shaffer et al. 2000). Approximately 50% of DLBCL express Bcl-6 in a variable proportion of tumor cells (Fig. 3 and 4A) (Skinnider et al. 1999; King et al. 2000; Artiga et al. 2002; Pileri et al. 2002; Braaten et al. 2003; Colomo et al. 2003; de Leval and Harris, 2003; Chang et al. 2004). Bcl-6 expression in DLBCL may be a signature of a GC differentiation stage of the original B-cell before malignant transformation, or may be turned on due to translocations involving the *BCL6* locus at 3q27 with variable partners of either the *Ig* family or *non-Ig* genes, or due to mutations in the 5′ non-translated regulatory region (Ye et al. 1993; Lo Coco et al. 1994; Ye et al. 1995; Pescarmona et al. 1997; Kramer et al. 1998; Skinnider et al. 1999; Capello et al. 2000; Butler et al. 2002; Falini and Mason, 2002; Pasqualucci et al. 2003; Ohno, 2006). It is likely that only co-expression of bcl-6 with CD10 reflects a true GC DLCBL derivation (Dogan et al. 2000; King et al. 2000; see Paragraph on expression of CD10). Indeed, bcl-6 and CD10 expression cluster together in DLBCL (Fig. 3). While bcl-6 protein or mRNA expression in DLBCL has been found to predict favorable outcomes by some investigators, this has not been confirmed by others (Lossos et al. 2001; Braaten et al. 2003; Colomo et al. 2003; Tzankov et al. 2003a; Chang et al. 2004; Winter et al. 2006). The same contradictory results have been found for *BCL6* rearrangements, which some authors report to be associated with a favorable outcome and others report no distinct prognostic significance (Lo Coco, 1994; Offit et al. 1994; Pescarmona et al. 1997; Kramer et al. 1998; Barrans et al. 2002a; Jerkeman et al. 2004). This controversy can be explained by the fact that translocations, leading to *non-Ig/BCL6* fusion products, indicate a poor prognosis, while those leading to *Ig/BCL6* fusions do not (Akasaka et al. 2000; Ueda et al. 2002; Ohno, 2006). *Bcl-6* mutations are probably also associated with distinct outcomes in DLBCL (Vitolo et al. 2002; Artiga et al. 2002).

CD10 is a membrane metalloproteinase expressed in GC B-cells (Shipp et al. 1989; King et al. 2000). Approximately 35% of DLBCL express CD10 (Fig. 3 and 4B) (Dogan et al. 2000; King et al. 2000; Fabiani et al. 2002; de Leval and Harris 2003; Tzankov et al. 2003a; Hans et al. 2004), but the prognostic relevance of such expression is controversial. Some authors reported an association of the CD10^+^ phenotype with a significantly lower rate of complete remissions, but most studies showed CD10 expression to be a favorable prognostic factor in DLBCL (Xu et al. 2001; Oshima et al. 2001; Uherova et al. 2001; Fabiani et al. 2002; Colomo et al. 2003; Biasoli et al. 2005; Muris et al. 2006b). A large proportion of CD10^+^ DLBCL express Bcl-6 (Fig. 3), indicating a GC-origin, and this phenotype seems to be particularly predictive of a favorable outcome (Ree et al. 2001; Barrans et al. 2002b; Colomo et al. 2003; Huang et al. 2002; McCluggage et al. 2002; Tzankov et al. 2003a; Chang et al. 2004; Hans et al. 2004; Zinzani et al. 2005; Berglund et al. 2005; van Imhoff et al. 2006).

In normal B-cells, MUM1/IRF4 expression probably drives the final steps of intra-GC B-cell differentiation and initiates subsequent steps of maturation towards plasma cells. Thus, MUM1 can be detected by immunohistochemistry in a small percentage of Bcl-6^−^ GC B-cells, post-GC B-cells and plasma cells (Falini et al. 2000). In DLBCL, MUM1 is expressed in 50 to 75% of both Bcl-6^+^ and Bcl-6^−^ samples, and may reflect derivation from B-cells at a late GC or post-GC stage of differentiation (Fig. 3 and 4C) (Falini et al. 2000; Tsuboi et al. 2000; Natkunam et al. 2001; Pileri et al. 2002; Chang et al. 2004; Hans et al. 2004; Saez et al. 2004; Zinzani et al. 2005). Gene expression profile analyses showed that *MUM1* clustered within the group of genes expressed by activated B-cell like DLBCL (Alizadeh et al. 2000). Subsequent TMA studies demonstrated that expression of MUM1 in at least 30% of tumor cells was associated with a significantly worse outcome (Chang et al. 2004; Hans et al. 2004; Tzankov et al. 2006; Muris et al. 2006b; van Imhoff et al. 2006), while other studies found no association between MUM1 expression and outcome (Colomo et al. 2003; Berglund et al. 2005).

FOXP1 (FORKHEAD BOX P1) is a transcription factor containing a forkhead DNA-binding domain (Kaestner et al. 2000). The *FOXP1* gene is located on chromosome 3 and is expressed in normal activated B-cells and in a subset of DLBCL with a predominantly non-GC phenotype (Fig. 3) (Barrans et al. 2004; Hans et al. 2004; Banham et al. 2005; Wlodarska et al. 2005). FOXP1 expression correlates with poor survival in DLBCL patients (Barrans et al. 2004; Banham et al. 2005). Interestingly, FOXP1 can be also detected in marginal zone B-cell lymphomas. Studies on the molecular mechanisms underlying FOXP1 expression in both DLBCL and MZL showed that its expression can result from a translocation t(3;14) (p13; q32) in 1% of cases, or may be related to an increased gene copy number, since 60% of FOXP1^+^ DLBCL harbor the trisomy 3 (Wlodarska et al. 2005; Fenton et al. 2006). Interestingly, 45% of FOXP1^+^ extranodal marginal zone B-cell lymphomas also have trisomy 3, and FOXP1 expression correlates with poor survival (Sagaert et al. 2006).

CD44 is a family of cell surface adhesion glyco-proteins that act as receptors for hyaluronate. CD44 molecules play a key role in normal lymphocyte development, homing and activation and are important for tumor spread (Irving et al. 1998). CD44 exist in a variety of alternatively spliced isoforms. Normal lymphocytes express the standard CD44 isoform (CD44s). In addition to CD44s, DLBCL may express larger splicing variants (CD44v), especially those containing exon v6/7, which are associated with disseminated malignancies in experimental models (Drillenburg and Pals 2000). Expression of CD44s and/or CD44v6 has been associated with shortened survival in DLBCL and clustered in bcl-6^−^ (non-GC) cases (Ristamaki et al. 1995; Drillenburg et al. 1999; Inagaki et al. 1999; Tzankov et al. 2003a).

A few studies point to the CD5 expression in DLBCL (Harada et al. 1999; Kroft et al. 2000; Suguro 2006). Its finding in 109 *de novo* cases was supposed to represent a unique subgroup of DLBCL because of the uniform phenotype (CD5^+^/CD10^−^/CD19^+^/CD20^+^/CD21^+^/CD23^−^/cyclin D1^−^), usual centroblastic morphology and aggressive clinical behavior (Yamaguchi et al. 2002; Suguro et al. 2006). However, the putative adverse prognostic relevance of CD5 expression has not been confirmed by other studies, which instead correlated CD5 positivity to the occurrence of other specific molecular aberrations (Katzenberger et al. 2002; Karnan et al. 2004; Zimpfer et al. 2004; Yoshioka et al. 2005).

VS38c and CD138 are markers of late post-GC differentiation and are often expressed in HIV-associated-, plasmablastic-, primary effusion- and ALK^+^ lymphomas (Delecluse et al. 1997; Delsol et al. 1997; Gatter and Warnke, 2001; Carbone and Gloghini, 2005; Teruya-Feldstein 2005; Levine 2006). Common DLBCL are rarely reactive with these markers (Pileri et al. 2002; de Leval and Harris, 2003).

Considering the expression of bcl-6, CD10 and MUM1 as well as CD44, CD138, bcl-2 and other biomarkers, different algorithms to identify GC and non-CG DLBCL have been proposed (e.g. Barrans et al. 2002b; Colomo et al. 2003; Tzankov et al. 2003a; Chang et al. 2004; Hans et al. 2004; Zinzani et al. 2005, Tzankov et al. 2006; Muris et al. 2006b; Oh et al. 2006; van Imhoff et al. 2006), but confirming the relevance of most of them is hampered by failures in results reproducibility and low validity.

## Prognostic Significance of Immunophenotypic Cell Cycle-and Apoptosis-controlling Proteins in DLBCL (Table 1)

Disruption of the physiological balance between cell proliferation and cell death is a universal feature of malignant tumors. Two major concurrent regulatory pathways control the cell cycle: The *p53* pathway, which regulates apoptosis and arrest in the G_1_-phase of the cell cycle, and the retinoblastoma (Rb) pathway, which regulates the G_1_-S transition. Cell cycle progression is regulated by a complex molecular network involving cyclins (CCN), cyclin-dependent kinases (CDK) and CDK inhibitors (CDKI). Genetic alterations and/or deregulations of many of these factors are frequently detected in DLBCL (Sherr 2000).

*p53,* one of the most frequently mutated genes in human cancer, monitors DNA integrity by arresting cells at the G_1_-phase or programming them to cell death when DNA is defective (Somasundaram 2000). *p53* is usually immunohistochemically undetectable in normal cells because of its rapid degradation. Missense mutations of *p53* usually result in protein stabilization, making it detectable by immunohistochemistry, but the absence of *p53* expression cannot be regarded as an unequivocal sign of a wild-type gene, since rare nonsense or frameshift mutations produce rapidly degradable *p53* proteins that fail to accumulate (Soussi and Beroud 2001). In DLBCL, *p53* is immunohistochemically detectable in 30 to 40% of cases, but only a fraction of *p53*^+^ DLBCL have an underlying mutation, thus *p53* mutational status can obviously not be deduced from immunohistochemically detected *p53* expression alone. Importantly, only *p53* mutations, which are found in about 20% of DLBCL, appear to be associated with clinical drug resistance and poor outcome (Villuendas et al. 1993; Piris et al. 1994; Kramer et al. 1996; Ichikawa et al. 1997; Koduru et al. 1997; Wilson et al. 1997; Moller et al. 1999; Llanos et al. 2001; Leroy et al. 2002; Kerbauy et al. 2004). The combined analysis of *p53* and its downstream target, p21, comprises the distinction between *p53* immunopositivity associated with *p53* mutation (*p53*^+^/p21^−^) and that, reflecting accumulation of wild-type *p53* (*p53*^+^/p21^+^) (Villuendas et al. 1993; Chilosi et al. 1996; Moller et al. 1999). Some studies showed that the *p53*^+^/p21^−^ (Δ*p53*) immunophenotype, used as a surrogate for *p53* mutations, is associated with treatment failure and poor survival in DLBCL as well, particularly in GC DLBCL (Moller et al. 1999; Pagano et al. 2001; Visco et al. 2006). We recently performed a TMA-based study on 297 DLBCL considering the prognostic significance Δ*p53* (Went et al. 2004), which was found in 21% of cases. In a multivariate model, high IPI and Δ*p53* were independent prognostic markers of poor survival.

Bcl-2, a mitochondrial inner membrane anti-apoptotic protein (Hockenbery et al. 1990), should be particularly discussed, because its prognostic importance in DLBCL has been confirmed by numerous studies (Moni et al. 1999; Rantanen et al. 2001; Shivakumar and Armitage, 2006) and bcl-2 associated treatment resistance can be abolished by the addition of rituximab to CHOP-therapy regimens (Mounier et al. 2003; Coiffier 2005). Bcl-2 is widely expressed in normal lymphoid tissues, but is absent in GC B-cells (Pezzella et al. 1990). The exemplary t(14;18) (q32; q21) translocation characteristic of follicular lymphoma (Tsujimoto et al. 1985), which induces production of high levels of bcl-2 protein, is observed in about 25% of DLBCL, but bcl-2 protein expression is found in >50% of DLBCL (Fig. 3) (Jacobson et al. 1993; Piris et al. 1994; Dalla-Favera et al. 1994; Gascoyne et al. 1997a; Pescarmona et al. 1997; Kramer et al. 1998; Skinnider et al. 1999; Rantanen et al. 2001; Huang et al. 2002; McCluggage et al. 2002; de Leval and Harris 2003; Barrans et al. 2003; Tzankov et al. 2003a; Iqbal et al 2004). Indeed, in the absence of BCL-2 translocation, amplification of 18q21 (containing the BCL2 gene) is another important mechanism for bcl-2 protein over-expression in DLBCL, and can be detected in about 30% of cases (Monni et al. 1997; Rao et al. 1998; Skinnider et al. 1999; Rantanen et al. 2001). Amplifications seem to be more frequent in non-GC DLBCL (18%) than GC DLBCL (5%), while the latter more frequently harbor the t(14;18) (q32; q21) (Huang et al. 2002; McCluggage et al. 2002; Rosenwald et al. 2002; Barrans et al. 2003; Iqbal et al. 2004; Kusumoto et al. 2005). There is no evidence that the presence of a BCL-2 translocation at diagnosis has any impact on the survival of patients with DLBCL, though the prognostic impact of bcl-2 protein expression, evaluated in multiple large-scale trials, is significant (Hill et al. 1996; Gascoyne et al. 1997a; Pescarmona et al. 1997; Bebb et al. 2002; Muris et al. 2006b). A recent publication supported the prognostic significance of t(14,18) in GC DLBCL (Barrans et al. 2003), while others suggest that bcl-2 expression may be of greater prognostic significance in non-GC DLBCL (Iqbal et al. 2006). The anti-apoptotic activity of bcl-2 is modulated in part by its ability to heterodimerize with bax, another member of the bcl-2 protein family with a pro-apoptotic activity (Oltvai et al. 1993). In two studies, low bax expression tended to be correlated with an adverse outcome in DLBCL (Gascoyne et al. 1997b; Sohn et al. 2003).

Survivin is a member of the apoptosis-inhibiting protein family and is expressed during mitosis, inhibiting apoptosis at the G_2_-M transition (Li and Ling, 2006). It is normally undetectable in adult tissues. In a large prospective DLBCL trial, survivin expression was detected in 60% of the cases and was an independent predictor of decreased survival (Adida et al. 2000). A second smaller study confirmed these observations for both phenotypical GC- and non-GC DLBCL (Watanuki-Miyauchi et al. 2005).

The monoclonal anti-Ki-67 antibody (MIB-1), which detects a protein expressed in the G_1_-, S-, G_2_- and M- but not G_0_-phases of the cell cycle, is widely used as a proliferation marker (Brown and Gatter, 2002). The functional significance of Ki-67 remains unclear (Scholzen and Gerdes, 2000). In DLBCL, the cell cycle fraction assessed by Ki-67 is variable, usually ranging from 30 to 100%, but is typically high (de Leval and Harris, 2003). A high proliferation index has been associated with an unfavorable clinical outcome in some studies, but not in others (Miller et al. 1994; Llanos et al. 2001; personal observations, (Fig. 3)). Since it has been suggested that Ki-67 plays a role in the ribosome biosynthesis rather than being directly responsible for cell proliferation (Scholzen and Gerdes, 2000), detecting markers directly involved in DNA replication might be a more precise method to evaluate the proliferative behavior of a tumor. The minichromosome maintenance (MCM) protein family, consisting of six abundant members of DNA-binding proteins, stands at the end of many signaling pathways involved in cell proliferation. MCMs ensure that synthesis of DNA is initiated only once during each cell cycle and are only expressed in cycling, but not in quiescent and differentiating cells (Tye, 1999). We recently demonstrated that expression of MCM2 in ≥40% of tumor cells is a negative prognostic marker for disease-specific survival in a large series of DLBCL (Obermann et al. 2005a).

**Figure 5:**
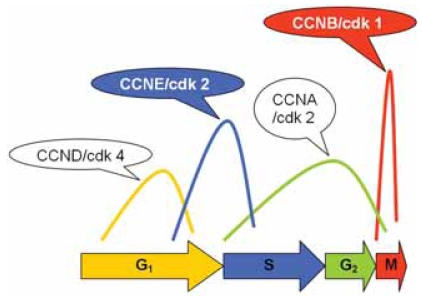
Schematic demonstration of cell cycle regulation. The most critically disturbed phases in diffuse large B-cell lymphomas are delineated in blue (G_1_-S transition) and red color (G_2_-M transition).

In normal cells, transition through the restriction point of the cell cycle in the G_1_-phase, beyond which cell proliferation is independent of external signaling, is negatively regulated by the Rb protein, which binds and inactivates E2F transcription factors whose activity is necessary for expression of S-phase genes. Under mitogenic stimulation, accumulation of D-type CCNs allows formation of active CDK4/CCND complexes that inactivate Rb, thus promoting E2F-mediated transcription and subsequent progression through the early (mitogen-dependent) G_1_-phase of the cell cycle. Later, CDK2/CCNE complexes drive the mitogen-independent G_1_-phase progression as well as the G_1_-S transition. CDK1/CCNB1 complexes play an important role in G_2_-M transition and execution of mitosis (Sherr 2000). Considering cell cycle regulation in DLBCL, expression of CCNB1, CCND2, CCND3, CCNE, CDK1 and CDK2 and CDKI p27 have been shown to be prognostically relevant (Erlanson et al. 1998; Saez et al. 1999; Sanchez-Beato et al. 1999; Ferreri et al. 2001; Moller et al. 2001; Filipits et al. 2002; Kuttler et al. 2002; Lin et al. 2003; Saez et al. 2004; Hans et al. 2005; Obermann et al. 2005b; Tzankov et al. 2006). Expression of CCND2 in more than 30% or of CCND3 in more than 50% of neoplastic cells, respectively, seems to predict inferior overall survival (Filipits et al. 2002; Hans et al. 2004 and 2005). In addition to the direct activation of CDK4, CCND3 can further promote cell proliferation by the sequestration of p27 and indirect activation of CDK2/CCNE, which might explain why high p27 expression (probably sequestered by CCND3) is an adverse prognostic factor in DLBCL (Saez et al. 1999; Sanchez-Beato et al. 1999; Lin et al. 2003). We recently showed that 35% of DLBCL express CCNE in >20% of tumor cells (Fig. 4D) despite the general lack of CCNE gene amplification (Tzankov et al. 2006), a constellation similar to that in classical Hodgkin lymphoma (Tzankov et al. 2003b). In classical Hodgkin lymphoma, CCNE over-expression seems to reflect profound deregulation of the cell cycle in Hodgkin and Reed-Sternberg cells (Tzankov et al. 2003b; Tzankov et al. 2005b) and has no prognostic significance, while CCNE in DLBCL obviously preserves its oncogenic potential to promote G_1_-S transition independent of extracellular mitogenic stimuli (Gong et al. 1995; Bortner and Rosenberg 1997). CCNE expression in >20% of tumor cells is an IPI-independent prognostic factor for both overall- and disease-specific survival and a predictive factor for poor response to CHOP treatment regimens in DLBCL (Tzankov et al. 2006). Expression of CCNE did not correlate with proliferation as assessed by Ki-67 (Tzankov et al. 2006), in agreement with previous observations (Erlanson et al. 1998). Thus, mitogen-independent deregulation of the G_1_-S transition possibly plays a more important oncogenic role than proliferative activity. Interestingly in that context, we and others demonstrated that detection of >1% CCNB1 stainable cells in DLBCL is also a stage-independent negative prognostic factor (Kuttler et al. 2002; Obermann et al. 2005b). In summary, deregulation of the G_1_-S (CCNE/CDK2) and G_2_-M transitions (CCNB1/CDK1) are probably most critical for the malignant potential of DLBCL (Fig. 5).

## Implementation of Receiver Operating Characteristic (ROC) Curves and Area Under ROC (AUROC) to Determine Optimize Prognostic Cut-Off Values of Immunophenotypic Markers in DLBCL

One of the main obstacles for practical translation of the marker profiles reviewed herein is the considerable variation in criteria used by different investigators to classify positive and negative cases, as well as the fact that in some instances (e.g. Bcl-2 and Bcl-6), the multiple mechanisms driving protein expression have not been taken into consideration. The quantity of positive cells and staining intensity for many of the phenotypic markers considered shows a continuous distribution from 0 to 100% in DLBCL. Cut-off levels for the different markers have a broad range and their sensitivity and specificity have not yet been critically addressed (Table 1).

**Figure 6:**
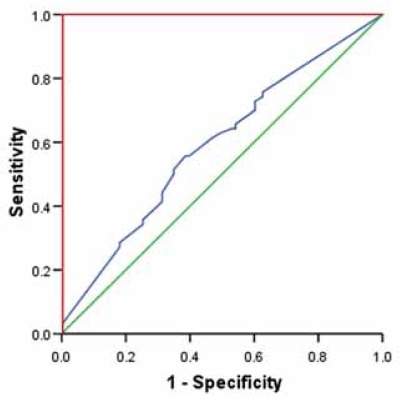
Typical receiver operating characteristics (ROC)-curve of a prognostic marker in diffuse large B-cell lymphoma (DLBCL) (blue), in that particular case FOXP1. The area under the ROC-curve is 0.583, p = 0.015, suggesting that FOXP1 determination in DLBCL is of significant prognostic importance. The optimal cut-off value for FOXP1 expression considering survival determined by ROC and Youden’s transformation was at 47,5% positivity (arrow) with a specificity of 59% and sensitivity of 57%; note that the cut-off point indicated by the arrow is as next to the coordinates 0.0;1.0. The reference diagonal green line corresponds to a variable without diagnostic capability. The ideal ROC-curve is delineated in red.

When a diagnostic test is based on a continuous variable, a range of different cut-off values may be investigated to decide which value should be used to discriminate between patients according to outcome (Bewick et al. 2004). In most instances, it is desirable to choose a test that has highest possible values for both sensitivity and specificity. A graphic of sensitivity against 1 – specificity is called a receiver operating characteristic (ROC) curve (Fig. 6). A perfect test would have a sensitivity and specificity both equal to 1. The ROC curve would start at coordinates X0; Y0, go vertically up the y-axis and then horizontally across to coordinates X1; Y1. A good test would be somewhere close to this ideal (Bewick et al. 2004). If a variable has no diagnostic or prognostic value, then a test based on it would be equally random and the ROC curve would run diagonally (Fig. 6). The performance of a diagnostic variable can be quantified by calculating the area under the ROC curve (AUROC). The ideal test would have an AUROC of 1, whereas a random guess would have an AUROC of 0.5. If there is no particular requirement on the sensitivity and specificity of a test, then the Youden’s index (Y) may be used to choose an appropriate cut-off for the descriptive values from the ROC curve:

Y=sensitivity+specificity-1 (Perkins and Schisterman,2006).

The maximum value Y can attain is 1, when the test is perfect. The coordinates from the ROC curve can be easily calculated and sorted by this index. Optimal cut-off values then should be determined with reference to Y nearest to 1. The potentials of implementation of ROC/AUROC for diagnostic purposes in immunohistochemistry have only recently been realized and addressed (e.g. Obermann et al. 2005a; Obermann et al. 2005b; Zlobec et al. 2006).

**Figure 7:**
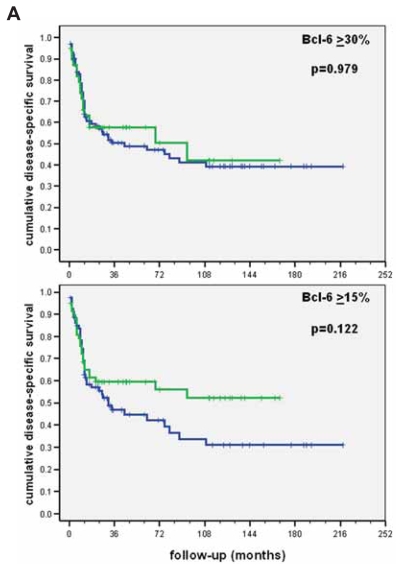
Comparison of results for the prognostic value of Bcl-6 in our diffuse large B-cell lymphoma series linked to disease-related mortality by the Kaplan-Meier method applying cut-off levels suggested in the literature (upper) and the ROC curves (lower).

Taking into consideration these statistic operators, we critically re-evaluated our own TMA series of 301 primary DLBCL (Tzankov et al. 2003a; Went et al. 2004; Zinzani et al. 2005; Tzankov et al. 2005a; Tzankov et al. 2006) to determine the optimal cut-off values for differentiation-associated antigen expression (Table 2). We linked the results to the clinical end-point “disease-related mortality” and critically compared the outcomes obtained considering the cut-off levels form the ROC curves and Y with those suggested in the literature. Comparison of the results linked to disease-related mortality by the Kaplan-Meier method for every factor unequivocally showed the superior discriminating power (increased sensitivity and specificity) of the cut-off levels calculated considering the ROC curves and Y (e.g. Bcl-6, Fig. 7). Furthermore, we compared the results on the molecular classification of our DLBCL series according to the “Hans’ algorithm” (Hans et al. 2004) using cut-off values of the variables from the original publication and those suggested by the ROC curves and Y. The Kaplan-Meier analysis showed a superior prognostic value of the phenotypic DLBCL classification according to the cut-off values from the ROC curves (data not shown).

## Perspectives

A high number of strong candidate biomarkers, particularly expressed proteins, that contribute to prognosis in DLBCL have been identified but not yet translated to practical utility mainly because of contradictory reports in the literature resulting from small sample sizes, referral and selection biases, and variable methodologies and cut-off levels used to determine positivity. These obstacles must be addressed before these biomarkers can be introduced into clinical practice. First, biomarker assessment in DLBCL should be standardized and validated applying powerful statistical methods. Second, the clinical material required to study such questions should be clearly documented and brought into TMAs, which should become an integral part of all clinical trials. The combination of tumor-specific biomarkers with patient-specific clinical factors in new predictive and prognostic models will enable successful individual risk-adjusted patient treatment.

## Figures and Tables

**Table 1:** Review of prognostic phenotypic markers in diffuse large B-cell lymphoma; cut-off levels according to varying reports in the literature. Note the broad range of cut-off levels applied.

Protein	Outcome*	Mechanism	Cut-off**
Bax	favorable	reciprocal relationship to Bcl-2	>50%
Bcl-2	unfavorable	anti-apoptosis, drug resistance	>10 to >50%
Bcl-6	favorable/uncertain	repression of post-GC gene transcription, reciprocal to MUM-1	>10 to >30%
CD5	unfavorable/uncertain	distinct cellular origin	>10 to >20%
CD10	favorable	signature of GC-phenotype	>10 to >30%
CD44s & v6	unfavorable	tumor dissemination promotion	>20 to >80%
CDK1 & 2	unfavorable	cell cycle promotion	>50 to >80%
CCNB1	unfavorable	facilitation of G_2_-M transition	>1%
CCND2	unfavorable	mitogen-dependent cell cycle progression	>30%
CCND3	unfavorable		>5 to >50%
CCNE	unfavorable	mitogen-independent cell cycle progression, chromosomal instability	>2 to >80%
FOXP1	unfavorable	transcriptional repressor in activated B-cells	>30 to 100%
Ki-67	uncertain	unknown	<60, >50, >65, >85%
Mcm2	unfavorable	efficient proliferation	>40%
Mdm2	unfavorable	increased degradation of p53	>10%
MUM-1	unfavorable	promotion of post-GC gene transcription, reciprocal to Bcl-6	>30%
p21	probably favorable	should be interpreted together with p53	>10%
p27	unfavorable	inactivation by CCND3 or SKP2	>5 to >15%
p53	unfavorable/uncertain	inactivation → lost cell cycle control	>5 to >20%
pRb	unfavorable	inactivation → cell cycle progression	>80%
SKP2	unfavorable	G_1_-S-transition by degradation of p27	>60 to >80%
Survivin	unfavorable	inhibition of apoptosis at G_2_-M	>10%

* indicates the mainstream finding of the reviewed studies.

** references from which data arose are mentioned in the according manuscript sections dealing with the individual markers.

**Table 2:** Determination of optimal cut-off levels considering disease-specific survival (DSS) for selected phenotypic markers and clinical parameters in our own series of diffuse large B-cell lymphomas applying receiver operating characteristic curves (ROC). The significance of each biomarker considering DSS was finally tested after dichotomization by the Kaplan-Meier (K-M) method. Factors in which values > cut-off have adverse prognostic effects are italicized.

Factor	AUROC	p-value^ROC^	Optimal cut-off^DSS^	Sensitivity	Specificity	p-value^DSS(K-M)^
*Bcl-2*	0.552	0.118	57.5% (60%)	57%	57%	0.082
Bcl-6	0.542	0.207	14.0% (15%)	49%	64%	0.122
CD10	0.538	0.199	7.5% (10%)	32%	77%	0.184
FOXP1	0.583	0.015	47.5% (50%)	57%	59%	0.014
MUM1	0.513	0.383	64.5% (65%)	52%	79%	0.007
LDH	0.593	0.027	295 U/L	63%	54%	<0.001
Ki-67	0.537	0.195	72.5% (75%)	42%	70%	0.122
age	0.599	<0.0001	65.5 (66)	61%	56%	0.005

## References

[b1-bmi-2007-403] Adida C Haioun C Gaulard P 2000 Prognostic significance of survivin expression in diffuse large B-cell lymphoma Blood 96 1921 5 10961895

[b2-bmi-2007-403] Akasaka T Ueda C Kurata M 2000 Nonimmunoglobulin (non-Ig)/BCL6 gene fusion in diffuse large B-cell lymphoma results in worse prognosis than Ig/BCL6 Blood 96 2907 11023530

[b3-bmi-2007-403] Alizadeh AA Eisen MB Davis RE 2000 Distinct types of diffuse large B-cell lymphoma identified by gene expression profiling Nature 403 503 11 10676951 10.1038/35000501

[b4-bmi-2007-403] Artiga M Saez A Romero C 2002 A short mutational hot spot in the first intron of BCL-6 is associated with increased BCL-6 expression and with longer overall survival in large B-cell lymphomas Am J Pathol 160 1371 80 11943722 10.1016/S0002-9440(10)62564-3 PMC1867208

[b5-bmi-2007-403] Banham AH Connors JM Brown PJ 2005 Expression of the FOXP1 transcription factor is strongly associated with inferior survival in patients with diffuse large B-cell lymphoma Clin Cancer Res 11 1065 72 15709173

[b6-bmi-2007-403] Barrans SL O’Connor SJ Evans PA 2002a Rearrangement of the BCL6 locus at 3q27 is an independent poor prognostic factor in nodal diffuse large B-cell lymphoma Br J Haematol 117 322 32 11972514 10.1046/j.1365-2141.2002.03435.x

[b7-bmi-2007-403] Barrans SL Carter I Owen R 2002b Germinal center phenotype and bcl-2 expression combined with the International Prognostic index improves patient risk stratification in diffuse large B-cell lymphoma Blood 99 1136 43 11830458 10.1182/blood.v99.4.1136

[b8-bmi-2007-403] Barrans SL Evans PA O’Connor SJ 2003 The t(14;18) is associated with germinal center-derived diffuse large B-cell lymphoma and is a strong predictor of outcome Clin Cancer Res 9 2133 9 12796378

[b9-bmi-2007-403] Barrans SL Fenton JA Banham A 2004 Strong expression of FOXP1 identifies a distinct subset of diffuse large B-cell lymphoma (DLBCL) patients with poor outcome Blood 104 2933 5 15238418 10.1182/blood-2004-03-1209

[b10-bmi-2007-403] Berglund M Thunberg U Amini RM 2005 Evaluation of immunophenotype in diffuse large B-cell lymphoma and its impact on prognosis Mod Pathol 18 1113 20 15920553 10.1038/modpathol.3800396

[b11-bmi-2007-403] Bewick V Cheek L Ball J 2004 Statistics review 13: Receiver operating characteristic curves Crit Care 8 508 12 15566624 10.1186/cc3000 PMC1065080

[b12-bmi-2007-403] Biasoli I Morais JC Scheliga A 2005 CD10 and Bcl-2 expression combined with the International Prognostic Index can identify subgroups of patients with diffuse large-cell lymphoma with very good or very poor prognoses Histopathology 46 328 33 15720419 10.1111/j.1365-2559.2005.02099.x

[b13-bmi-2007-403] Bortner DM Rosenberg MP 1997 Induction of mammary gland hyperplasia and carcinomas in transgenic mice expressing human cyclin E Mol Cell Biol 17 453 9 8972226 10.1128/mcb.17.1.453 PMC231770

[b14-bmi-2007-403] Braaten K Betensky R de Leval L 2003 Bcl-6 expression predicts improved survival in patients with primary central nervous system lymphoma Clin Cancer Res 9 1063 9 12631608

[b15-bmi-2007-403] Brown DC Gatter KC 2002 Ki67 protein: the immaculate deception? Histopathology 40 2 11 11903593 10.1046/j.1365-2559.2002.01343.x

[b16-bmi-2007-403] Butler M Lida S Capello D 2002 Alternative translocation breakpoint cluster region 5′ to BCL6 in B-cell non-Hodgkin’s lymphoma Cancer Res 62 4089 94 12124346

[b17-bmi-2007-403] Capello D Vitolo U Pasqualucci L 2000 Distribution and pattern of BCL-6 mutations throughout the spectrum of B-cell neoplasia Blood 95 651 9 10627476

[b18-bmi-2007-403] Carbone A Gloghini A 2005 AIDS-related lymphomas: from pathogenesis to pathology Br J Haematol 130 662 70 16115121 10.1111/j.1365-2141.2005.05613.x

[b19-bmi-2007-403] Cattoretti G Chang CC Cechova K 1995 BCL-6 protein is expressed in germinal-center B-cells Blood 86 45 53 7795255

[b20-bmi-2007-403] Chang CC McClintock S Cleveland RP 2004 Immunohistochemical expression patterns of germinal center and activation B-cell markers correlate with prognosis in diffuse large B-cell lymphoma Am J Surg Pathol 28 464 70 15087665 10.1097/00000478-200404000-00005

[b21-bmi-2007-403] Chilosi M Doglioni C Magalini A 1996 p21/WAF1 cyclinkinase inhibitor expression in non-Hodgkin’s lymphomas: a potential marker of *p53* tumor-suppressor gene function Blood 88 4012 20 8916968

[b22-bmi-2007-403] Coiffier B 2005 Treatment of diffuse large B-cell lymphoma Curr Hematol Rep 4 7 14 15610654

[b23-bmi-2007-403] Colomo L Lopez-Guillermo A Perales M 2003 Clinical impact of the differentiation profile assessed by immunophenotyping in patients with diffuse large B-cell lymphoma Blood 101 78 84 12393466 10.1182/blood-2002-04-1286

[b24-bmi-2007-403] Davis T Czerwinski K Levy R 1999 Therapy of B-cell lymphoma with anti-CD20 antibodies can result in the loss of CD20 antigen expression Clin Cancer Res 5 611 5 10100713

[b25-bmi-2007-403] De Leval L Harris NL 2003 Variability in immunophenotype in diffuse large B-cell lymphoma and its clinical relevance Histopathology 43 509 28 14636252 10.1111/j.1365-2559.2003.01758.x

[b26-bmi-2007-403] De Paepe P De Wolf-Peeters C 2007 Diffuse large B-cell lymphoma: a heterogeneous group of non-Hodgkin lymphomas comprising several distinct clinicopathological entities Leukemia 21 37 43 17039226 10.1038/sj.leu.2404449

[b27-bmi-2007-403] Delecluse H Anagnostopoulos I Dallenbach F 1997 Plasmablastic lymphomas of the oral cavity: a new entity associated with the human immunodeficiency virus infection Blood 89 1413 20 9028965

[b28-bmi-2007-403] Delsol G Lamant L Mariame B 1997 A new subtype of large B-cell lymphoma expressing the ALK kinase and lacking the 2; 5 translocation Blood 89 1483 90 9057627

[b29-bmi-2007-403] Dogan A Bagdi E Munson P 2000 CD10 and bcl-6 expression in paraffin sections of normal lymphoid tissue and B-cell lymphomas Am J Surg Pathol 24 846 52 10843287 10.1097/00000478-200006000-00010

[b30-bmi-2007-403] Drillenburg P Wielenga VJ Kramer MH 1999 CD44 expression predicts disease outcome in localized large B-cell lymphoma Leukemia 13 1448 55 10482998 10.1038/sj.leu.2401490

[b31-bmi-2007-403] Drillenburg P Pals ST 2000 Cell adhesion receptors in lymphoma dissemination Blood 95 1900 10 10706853

[b32-bmi-2007-403] Erlanson M Portin C Linderholm B 1998 Expression of cyclin E and the cyclin-dependent kinase inhibitor p27 in malignant lymphomas-prognostic implications Blood 92 770 7 9680343

[b33-bmi-2007-403] Fabiani B Delmer A Lepage E 2004 CD10 expression in diffuse large B-cell lymphomas does not influence survival Virchows Arch 445 545 51 15517363 10.1007/s00428-004-1129-7

[b34-bmi-2007-403] Falini B Pileri S Stein H 1990 Variable expression of leucocyte-common (CD45) antigen in CD30 (Ki1)-positive anaplastic large-cell lymphoma: implications for the differential diagnosis between lymphoid and nonlymphoid malignancies Hum Pathol 21 624 9 1693592 10.1016/s0046-8177(96)90009-x

[b35-bmi-2007-403] Falini B Fizzotti M Pucciarini A 2000 A monoclonal antibody (MUM1p) detects expression of the MUM1/IRF4 protein in a subset of germinal center B-cells, plasma cells and activated T-cells Blood 95 2084 92 10706878

[b36-bmi-2007-403] Falini B Mason DY 2002 Proteins encoded by genes involved in chromosomal alterations in lymphoma and leukemia: clinical value of their detection by immunocytochemistry Blood 99 409 26 11781220 10.1182/blood.v99.2.409

[b37-bmi-2007-403] Fenton JA Schuuring E Barrans SL 2006 t(3;14) (p14; q32) results in aberrant expression of FOXP1 in a case of diffuse large B-cell lymphoma Genes Chromosomes Cancer 45 164 8 16252263 10.1002/gcc.20278

[b38-bmi-2007-403] Ferreri A Ponzoni M Pruneri G 2001 Immunoreactivity for p27(KIP1) and cyclin E is an independent predictor of survival in primary gastric non-Hodgkin lymphoma Int J Cancer 94 599 604 11745451 10.1002/ijc.1509

[b39-bmi-2007-403] Filipits M Jaeger U Pohl G 2002 Cyclin D3 is a predictive and prognostic factor in diffuse large B-cell lymphoma Clin Cancer Res 8 729 33 11895902

[b40-bmi-2007-403] Gascoyne RD Adomat SA Krajewski S 1997a Prognostic significance of Bcl-2 protein expression and Bcl-2 gene rearrangement in diffuse aggressive non-Hodgkin’s lymphoma Blood 90 244 51 9207459

[b41-bmi-2007-403] Gascoyne R Krajewska M Krajewski S 1997b Prognostic significance of bax protein expression in diffuse aggressive non-Hodgkin’s lymphoma Blood 90 3173 8 9376600

[b42-bmi-2007-403] Gascoyne RD 2004 Emerging prognostic factors in diffuse large B-cell lymphoma Curr Opin Oncol 16 436 41 15314511 10.1097/00001622-200409000-00004

[b43-bmi-2007-403] Gatter KC Warnke RA Jaffe ES Harris NL Stein H Vardiman JW 2001 Diffuse large B-cell lymphoma Pathology and genetics of tumours of the haematopoietic and lymphoid system Lyon IARC Press 171 4

[b44-bmi-2007-403] Gong J Traganos F Darzynkiewicz Z 1995 Threshold expression of cyclin E but not D type cyclins characterizes normal and tumour cells entering S phase Cell Prolif 28 337 46 7626688 10.1111/j.1365-2184.1995.tb00075.x

[b45-bmi-2007-403] Hans CP Weisenburger DD Greiner TC 2004 Confirmation of the molecular classification of diffuse large B-cell lymphoma by immunohistochemistry using a tissue microarray Blood 103 275 82 14504078 10.1182/blood-2003-05-1545

[b46-bmi-2007-403] Hans CP Weisenburger DD Greiner TC 2005 Expression of PKC-beta or cyclin D2 predicts for inferior survival in diffuse large B-cell lymphoma Mod Pathol 18 1377 84 15920548 10.1038/modpathol.3800434

[b47-bmi-2007-403] Harada S Suzuki R Uehira K 1999 Molecular and immunological dissection of diffuse large B-cell lymphoma: CD5^+^, and CD5^−^ with CD10^+^ groups may constitute clinically relevant subtypes Leukemia 13 1441 7 10482997 10.1038/sj.leu.2401487

[b48-bmi-2007-403] Held G Poschel V Pfreundschuh M 2006 Rituximab for the treatment of diffuse large B-cell lymphomas Expert Rev Anticancer Ther 6 1175 86 16925484 10.1586/14737140.6.8.1175

[b49-bmi-2007-403] Hill ME MacLennan KA Cunningham DC 1996 Prognostic significance of BCL-2 expression and bcl-2 major breakpoint region rearrangement in diffuse large cell non-Hodgkin’s lymphoma: a British National Lymphoma Investigation Study Blood 88 1046 51 8704213

[b50-bmi-2007-403] Hockenbery D Nunez G Milliman C 1990 Bcl-2 is an inner mitochondrial membrane protein that blocks programmed cell death Nature 348 334 6 2250705 10.1038/348334a0

[b51-bmi-2007-403] Hsi ED 2001 The search for meaningful prognostic markers in diffuse large B-cell lymphoma Am J Clin Pathol 115 481 3 11293894 10.1309/N87Q-F48C-PU2R-JUX0

[b52-bmi-2007-403] Huang J Sanger W Greiner T 2002 The t(14;18) defines a unique subset of diffuse large B-cell lymphoma with a germinal center B-cell gene expression profile Blood 99 2285 90 11895757 10.1182/blood.v99.7.2285

[b53-bmi-2007-403] Ichikawa A Kinoshia T Watanabe T 1997 Mutations of the *p53* gene as a prognostic factor in aggressive B-cell lymphoma New Engl J Med 337 529 34 9262496 10.1056/NEJM199708213370804

[b54-bmi-2007-403] Inagaki H Banno S Wakita A 1999 Prognostic significance of CD44v6 in diffuse large B-cell lymphoma Mod Pathol 12 546 52 10349995

[b55-bmi-2007-403] Iqbal J Sanger WG Horsman DE 2004 BCL2 translocation defines a unique tumor subset within the germinal center B-cell-like diffuse large B-cell lymphoma Am J Pathol 165 159 66 15215171 10.1016/s0002-9440(10)63284-1 PMC1618550

[b56-bmi-2007-403] Iqbal J Neppalli VT Wright G 2006 BCL2 expression is a prognostic marker for the activated B-cell-like type of diffuse large B-cell lymphoma J Clin Oncol 24 961 8 16418494 10.1200/JCO.2005.03.4264

[b57-bmi-2007-403] Irving JA Cain G Howard M 1998 The role of alternative splicing of the adhesion molecule, CD44, in lymphoid malignancy J Clin Pathol 51 776 80 10023342 10.1136/jcp.51.10.776 PMC500934

[b58-bmi-2007-403] Jacobson J Wilkes B Kwaiatkowski D 1993 Bcl-2 rearrangements in *de novo* diffuse large cell lymphoma. Association with distinctive clinical features Cancer 72 231 36 8508412 10.1002/1097-0142(19930701)72:1<231::aid-cncr2820720141>3.0.co;2-5

[b59-bmi-2007-403] Jerkeman M Aman P Cavallin-Stahl E 2002 Prognostic implications of BCL6 rearrangement in uniformly treated patients with diffuse large B-cell lymphoma—a Nordic Lymphoma Group study Int J Oncol 20 161 5 11743658 10.3892/ijo.20.1.161

[b60-bmi-2007-403] Karnan S Tagawa H Suzuki R 2004 Analysis of chromosomal imbalances in de novo CD5-positive diffuse large-B-cell lymphoma detected by comparative genomic hybridization Genes Chromosomes Cancer 39 77 81 14603444 10.1002/gcc.10298

[b61-bmi-2007-403] Katzenberger T Lohr A Schwarz S 2003 Genetic analysis of *de novo* CD5^+^ diffuse large B-cell lymphomas suggests an origin from a somatically mutated CD5^+^ progenitor B-cell Blood 101 699 702 12393524 10.1182/blood-2002-06-1726

[b62-bmi-2007-403] Kerbauy FR Colleoni GW Saad ST 2004 Detection and possible prognostic relevance of *p53* gene mutations in diffuse large B-cell lymphoma. An analysis of 51 cases and review of the literature Leuk Lymphoma 45 2071 8 15370252 10.1080/10428190410001713170

[b63-bmi-2007-403] King BE Chen C Locker J 2000 Immunophenotypic and genotypic markers of follicular center cell neoplasia in diffuse large B-cell lymphoma Mod Pathol 13 1219 31 11106080 10.1038/modpathol.3880226

[b64-bmi-2007-403] Kramer MH Hermans J Parker J 1996 Clinical significance of bcl-2 and *p53* protein expression in diffuse large B-cell lymphoma: a population-based study J Clin Oncol 14 2131 8 8683246 10.1200/JCO.1996.14.7.2131

[b65-bmi-2007-403] Kramer MH Hermans J Wijburg E 1998 Clinical relevance of BCL2, BCL6, and MYC rearrangements in diffuse large B-cell lymphoma Blood 92 3152 62 9787151

[b66-bmi-2007-403] Kroft S Howard M Picker L 2000 *De novo* CD5+ diffuse large B-cell lymphomas—a heterogeneous group containing an unusual form of splenic lymphoma Am J Clin Pathol 114 523 33 11026098 10.1309/RM1Q-1T0B-WKQB-AF5A

[b67-bmi-2007-403] Kusumoto S Kobayashi Y Sekiguchi N 2005 Diffuse large B-cell lymphoma with extra Bcl-2 gene signals detected by FISH analysis is associated with a “non-germinal center phenotype” Am J Surg Pathol 29 1067 73 16006802

[b68-bmi-2007-403] Kuttler F Valnet-Rabier MB Angonin R 2002 Relationship between expression of genes involved in cell cycle control and apoptosis in diffuse large B-cell lymphoma: a preferential survivincyclin B link Leukemia 16 726 35 11960356 10.1038/sj.leu.2402427

[b69-bmi-2007-403] Leroy K Haioun C Lepage E 2002 *p53* gene mutations are associated with poor survival in low and low-intermediate risk diffuse large B-cell lymphoma Ann Oncol 13 1108 15 12176791 10.1093/annonc/mdf185

[b70-bmi-2007-403] Levine AM 2006 AIDS-related lymphoma Semin Oncol Nurs 22 80 9 16720230 10.1016/j.soncn.2006.01.004

[b71-bmi-2007-403] Li F Ling X 2006 Survivin study: an update of “what is the next wave”? J Cell Physiol 208 476 86 16557517 10.1002/jcp.20634 PMC2821201

[b72-bmi-2007-403] Lin Z Lim S Lim MS 2003 Growth regulation by p27^Kip1^ is abrogated by multiple mechanisms in aggressive malignant lymphomas Br J Haematol 121 739 48 12780788 10.1046/j.1365-2141.2003.04354.x

[b73-bmi-2007-403] Llanos M Alvarez-Arguelles H Aleman R 2001 Prognostic significance of Ki-67 nuclear proliferative antigen, bcl-2 protein, and *p53* expression in follicular and diffuse large B-cell lymphoma Med Oncol 18 15 22 11778965 10.1385/MO:18:1:15

[b74-bmi-2007-403] Lo Coco F Ye BH Lista F 1994 Rearrangements of the BCL6 gene in diffuse large cell non-Hodgkin’s lymphoma Blood 83 1757 9 8142643

[b75-bmi-2007-403] Lossos IS Jones C Warnke R 2001 Expression of a single gene, BCL-6, strongly predicts survival in patients with diffuse large B-cell lymphoma Blood 98 945 51 11493437 10.1182/blood.v98.4.945

[b76-bmi-2007-403] Lossos IS 2005 Molecular pathogenesis of diffuse large B-cell lymphoma J Clin Oncol 23 6351 7 16155019 10.1200/JCO.2005.05.012

[b77-bmi-2007-403] McCluggage WG Catherwood M Alexander HD 2002 Immunohistochemical expression of CD10 and t(14;18) chromosomal translocation may be indicators of follicle centre cell origin in nodal diffuse large B-cell lymphoma Histopathology 41 414 20 12405909 10.1046/j.1365-2559.2002.01463.x

[b78-bmi-2007-403] Miller T Grogan T Dahlberg S 1994 Prognostic significance of the KI-67-associated proliferative antigen in aggressive non-Hodgkin’s lymphomas: a prospective Southwest Oncology Group trial Blood 83 1460 6 8123837

[b79-bmi-2007-403] Mitterlechner T Fiegl M Muhlbock H 2006 Epidemiology of non-Hodgkin lymphomas in Tyrol/Austria from 1991 to 2000 J Clin Pathol 59 48 55 16394280 10.1136/jcp.2005.026815 PMC1860250

[b80-bmi-2007-403] Moller M Gerdes A Skjodt K 1999 Disrupted p53 function as predictor of treatment failure and poor prognosis in B- and T-cell non-Hodgkin’s lymphoma Clin Cancer Res 5 1085 91 10353742

[b81-bmi-2007-403] Moller MB Nielsen O Pedersen NT 2001 Cyclin D3 expression in non-Hodgkin lymphoma. Correlation with other cell cycle regulators and clinical features Am J Clin Pathol 115 404 12 11242797 10.1309/8KF0-0Y0C-2F4L-UHXL

[b82-bmi-2007-403] Monni O Joensuu H Franssila K 1997 BCL2 overexpression associated with chromosomal amplification in diffuse large B-cell lymphoma Blood 90 1168 74 9242549

[b83-bmi-2007-403] Morgensztern D Lossos IS 2005 Molecular prognostic factors in diffuse large B-cell lymphoma Curr Treat Options Oncol 6 269 77 15967080 10.1007/s11864-005-0031-0

[b84-bmi-2007-403] Mounier N Briere J Gisselbrecht C 2003 Rituximab plus CHOP (R-CHOP) overcomes bcl-2-associated resistance to chemotherapy in elderly patients with diffuse large B-cell lymphoma (DLBCL) Blood 101 4279 84 12576316 10.1182/blood-2002-11-3442

[b85-bmi-2007-403] Muris JJ Meijer CJ Ossenkoppele GJ 2006a Apoptosis resistance and response to chemotherapy in primary nodal diffuse large B-cell lymphoma Hematol Oncol 24 97 104 16715473 10.1002/hon.774

[b86-bmi-2007-403] Muris JJ Meijer CJ Vos W 2006b Immunohistochemical profiling based on Bcl-2, CD10 and MUM1 expression improves risk stratification in patients with primary nodal diffuse large B-cell lymphoma J Pathol 208 714 23 16400625 10.1002/path.1924

[b87-bmi-2007-403] Natkunam Y Warnke R Montgomery K 2001 Analysis of MUM1/IRF4 protein expression using tissue microarrays and immunohistochemistry Mod Pathol 14 686 94 11455001 10.1038/modpathol.3880373

[b88-bmi-2007-403] Obermann EC Went P Zimpfer A 2005a Expression of mini-chromosome maintenance protein 2 as a marker for proliferation and prognosis in diffuse large B-cell lymphoma: a tissue microarray and clinico-pathological analysis BMC Cancer 5 162 16368013 10.1186/1471-2407-5-162 PMC1343577

[b89-bmi-2007-403] Obermann EC Went P Pehrs AC 2005b Cyclin B1 expression is an independent prognostic marker for poor outcome in diffuse large B-cell lymphoma Oncol Rep 14 1461 7 16273239

[b90-bmi-2007-403] Offit K Lo Coco F Louie DC 1994 Rearrangement of the bcl-6 gene as a prognostic marker in diffuse large-cell lymphoma N Engl J Med 331 74 80 8208268 10.1056/NEJM199407143310202

[b91-bmi-2007-403] Oh YH Park CK 2006 Prognostic evaluation of nodal diffuse large B-cell lymphoma by immunohistochemical profiles with emphasis on CD138 expression as a poor prognostic factor J Korean Med Sci 21 397 405 16778379 10.3346/jkms.2006.21.3.397 PMC2729941

[b92-bmi-2007-403] Ohno H 2006 Pathogenetic and clinical implications of non-immunoglobulin; BCL6 translocations in B-cell non-Hodgkin’s lymphoma J Clin Exp Hematop 46 43 53 17142954 10.3960/jslrt.46.43

[b93-bmi-2007-403] Ohshima K Kawasaki C Muta K 2001 CD10 and Bcl10 expression in diffuse large B-cell lymphoma: CD10 is a marker of improved prognosis Histopathology 39 156 62 11493332 10.1046/j.1365-2559.2001.01196.x

[b94-bmi-2007-403] Oltvai ZN Milliman CL Korsmeyer SJ 1993 Bcl-2 heterodimerizes in vivo with a conserved homolog, Bax, that accelerates programmed cell death Cell 74 609 19 8358790 10.1016/0092-8674(93)90509-o

[b95-bmi-2007-403] Perkins NJ Schisterman EF 2006 The inconsistency of “optimal” cutpoints obtained using two criteria based on the receiver operating characteristic curve Am J Epidemiol 163 670 5 16410346 10.1093/aje/kwj063 PMC1444894

[b96-bmi-2007-403] Pasqualucci L Migliazza A Basso K 2003 Mutations of the BCL6 protooncogene disrupt its negative autoregulation in diffuse large B-cell lymphoma Blood 101 2914 23 12515714 10.1182/blood-2002-11-3387

[b97-bmi-2007-403] Pescarmona E De Sanctis V Pistilli A 1997 Pathogenetic and clinical implications of Bcl-6 and Bcl-2 gene configuration in nodal diffuse large B-cell lymphomas J Pathol 183 281 6 9422982 10.1002/(SICI)1096-9896(199711)183:3<281::AID-PATH1134>3.0.CO;2-Z

[b98-bmi-2007-403] Pezzella F Tse AG Cordell JL 1990 Expression of the bcl-2 oncogene protein is not specific for the 14;18 chromosomal translocation Am J Pathol 137 225 32 2201196 PMC1877598

[b99-bmi-2007-403] Pileri SA Went P Ascani S 2002 Diffuse large B-cell lymphoma: one or more entities? Present controversies and possible tools for its subclassification Histopathology 41 482 509 12460202 10.1046/j.1365-2559.2002.01538.x

[b100-bmi-2007-403] Piris M Pezella F Martinez-Montero J 1994 *p53* and bcl-2 expression in high-grade B-cell lymphomas: correlation with survival time Br J Cancer 69 337 41 8297731 10.1038/bjc.1994.61 PMC1968699

[b101-bmi-2007-403] Rantanen S Monni O Joensuu H 2001 Causes and consequences of bcl-2 overexpression in diffuse large B-cell lymphoma Leuk Lymphoma 42 1089 98 11697626 10.3109/10428190109097729

[b102-bmi-2007-403] Rao PH Houldsworth J Dyomina K 1998 Chromosomal and gene amplification in diffuse large B-cell lymphoma Blood 92 234 40 9639522

[b103-bmi-2007-403] Ree HJ Yang WI Kim CW 2001 Coexpression of Bcl-6 and CD10 in diffuse large B-cell lymphomas, significance of Bcl-6 expression patterns in identifying germinal center B-cell lymphoma Hum Pathol 32 954 62 11567225 10.1053/hupa.2001.27118

[b104-bmi-2007-403] Ristamaki R Joensuu H Soderstrom KO 1995 CD44v6 expression in non-Hodgkin’s lymphoma: an association with low histological grade and poor prognosis J Pathol 176 259 67 7545748 10.1002/path.1711760308

[b105-bmi-2007-403] Rosenwald A Wright G Chan W 2002 The use of molecular profiling to predict survival after chemotherapy for diffuse large-B-cell lymphoma N Engl J Med 25 1937 47 12075054 10.1056/NEJMoa012914

[b106-bmi-2007-403] Rosenwald A Staudt LM 2003 Gene expression profiling of diffuse large B-cell lymphoma Leuk Lymphoma 44 Suppl 3 S41 7 15202524 10.1080/10428190310001623775

[b107-bmi-2007-403] Saez A Sanchez E Sanchez-Beato M 1999 p27KIP1 is abnormally expressed in diffuse large B-cell lymphomas and is associated with an adverse clinical outcome Br J Cancer 80 1427 34 10424746 10.1038/sj.bjc.6690539 PMC2363083

[b108-bmi-2007-403] Saez AI Saez AJ Artiga MJ 2004 Building an outcome predictor model for diffuse large B-cell lymphoma Am J Pathol 164 613 22 14742266 10.1016/S0002-9440(10)63150-1 PMC1602255

[b109-bmi-2007-403] Sagaert X de Paepe P Libbrecht L 2006 Forkhead box protein P1 expression in mucosa-associated lymphoid tissue lymphomas predicts poor prognosis and transformation to diffuse large B-cell lymphoma J Clin Oncol 24 2490 7 16636337 10.1200/JCO.2006.05.6150

[b110-bmi-2007-403] Sanchez-Beato M Camacho F Martinez-Montero J 1999 Anomalous high p27/KIP1 expression in a subset of aggressive B-cell lymphomas is associated with cyclin D3 overexpression. p27/KIP1-cyclin D3 colocalization in tumor cells Blood 94 765 72 10397744

[b111-bmi-2007-403] Savage KJ 2006 Primary mediastinal large B-cell lymphoma Oncologist 11 488 95 16720849 10.1634/theoncologist.11-5-488

[b112-bmi-2007-403] Scholzen T Gerdes J 2000 The Ki-67 protein: from the known and the unknown J Cell Physiol 182 311 22 10653597 10.1002/(SICI)1097-4652(200003)182:3<311::AID-JCP1>3.0.CO;2-9

[b113-bmi-2007-403] Shaffer A Yu X He Y 2000 BCL-6 represses genes that function in lymphocyte differentiation, inflammation and cell cycle control Immunity 13 199 212 10981963 10.1016/s1074-7613(00)00020-0

[b114-bmi-2007-403] Sherr CJ 2000 The Pezcoller lecture: cancer cell cycles revisited Cancer Res 60 3689 95 10919634

[b115-bmi-2007-403] Shipp MA Vijayaraghavan J Schmidt EV 1989 Common acute lymphoblastic leukemia antigen (CALLA) is active neutral endopeptidase 24.11 (‘enkephalinase’): direct evidence by cDNA transfection analysis Proc Natl Acad Sci USA 86 297 301 2521388 10.1073/pnas.86.1.297 PMC286451

[b116-bmi-2007-403] Shipp MA Harrington DP Anderson JR 1993 A predictive model for aggressive NHL: The International non-Hodgkin’s Lymphoma Prognostic Factors Project N Engl J Med 329 987 94 8141877 10.1056/NEJM199309303291402

[b117-bmi-2007-403] Shivakumar L Armitage JO 2006 Bcl-2 gene expression as a predictor of outcome in diffuse large B-cell lymphoma Clin Lymphoma Myeloma 6 455 7 16796775 10.3816/CLM.2006.n.025

[b118-bmi-2007-403] Skinnider BF Horsman DE Dupuis B 1999 Bcl-6 and Bcl-2 protein expression in diffuse large B-cell lymphoma and follicular lymphoma: correlation with 3q27 and 18q21 chromosomal abnormalities Hum Pathol 30 803 8 10414499 10.1016/s0046-8177(99)90141-7

[b119-bmi-2007-403] Sohn SK Jung JT Kim DH 2003 Prognostic significance of bcl-2, bax, and p53 expression in diffuse large B-cell lymphoma Am J Hematol 73 101 7 12749011 10.1002/ajh.10333

[b120-bmi-2007-403] Somasundaram K 2000 Tumor suppressor p53: regulation and function Front Biosci 5 D424 37 10762600 10.2741/somasund

[b121-bmi-2007-403] Soussi T Beroud C 2001 Assessing TP53 status in human tumours to evaluate clinical outcome Nat Rev Cancer 1 233 40 11902578 10.1038/35106009

[b122-bmi-2007-403] Suguro M Tagawa H Kagami Y 2006 Expression profiling analysis of the CD5^+^ diffuse large B-cell lymphoma subgroup: development of a CD5 signature Cancer Sci 97 868 74 16856881 10.1111/j.1349-7006.2006.00267.x PMC11158556

[b123-bmi-2007-403] Teruya-Feldstein J 2005 Diffuse large B-cell lymphomas with plasmablastic differentiation Curr Oncol Rep 7 357 63 16091196 10.1007/s11912-005-0062-5

[b124-bmi-2007-403] Torlakovic E Torlakovic G Nguyen P 2002 The value of anti-Pax-5 immunostaining in routinely fixed and paraffin-embedded sections. A novel pan pre-B and B-cell marker Am J Surg Pathol 26 1143 50 10.1097/00000478-200210000-00011 12360049

[b125-bmi-2007-403] Tsuboi K Iida S Inagaki H 2000 MUM1/IRF4 expression as a frequent event in mature lymphoid malignancies Leukemia 14 449 56 10720141 10.1038/sj.leu.2401696

[b126-bmi-2007-403] Tsujimoto Y Cossman J Jaffe E 1985 Involvement of the bcl-2 gene in human follicular lymphoma Science 228 1440 3 3874430 10.1126/science.3874430

[b127-bmi-2007-403] Tye BK 1999 MCM proteins in DNA replication Annu Rev Biochem 68 649 86 10872463 10.1146/annurev.biochem.68.1.649

[b128-bmi-2007-403] Tzankov A Pehrs AC Zimpfer A 2003a Prognostic significance of CD44 expression in diffuse large B-cell lymphoma of activated and germinal centre B-cell-like types: a tissue microarray analysis of 90 cases J Clin Pathol 56 747 52 14514777 10.1136/jcp.56.10.747 PMC1770073

[b129-bmi-2007-403] Tzankov A Zimpfer A Lugli A 2003b High-throughput tissue microarray analysis of G1-cyclin alterations in classical Hodgkin’s lymphoma indicates overexpression of cyclin E1 J Pathol 199 201 7 12533833 10.1002/path.1279

[b130-bmi-2007-403] Tzankov A Went P Zimpfer A 2005a Tissue microarray technology: principles, pitfalls and perspectives—lessons learned from hematological malignancies Exp Gerontol 40 737 44 16125349 10.1016/j.exger.2005.06.011

[b131-bmi-2007-403] Tzankov A Zimpfer A Went P 2005b Aberrant expression of cell cycle regulators in Hodgkin and Reed-Sternberg cells of classical Hodgkin lymphoma Mod Pathol 18 90 6 15389259 10.1038/modpathol.3800276

[b132-bmi-2007-403] Tzankov A Gschwendtner A Augustin F 2006 Diffuse large B-cell lymphoma with overexpression of cyclin E substantiates poor standard treatment response and inferior outcome Clin Cancer Res 12 2125 32 16609025 10.1158/1078-0432.CCR-05-2135

[b133-bmi-2007-403] Ueda C Uchiyama T Ohno H 2002 Immunoglobulin (Ig)/BCL6 versus non-Ig/BCL6 gene fusion in diffuse large B-cell lymphoma corresponds to a high- versus low-level expression of BCL6 mRNA Blood 99 2624 5 11926184 10.1182/blood-2001-11-0117

[b134-bmi-2007-403] Uherova P Ross C Schnitzer B 2001 The clinical significance of CD10 antigen expression in diffuse large B-cell lymphomas Am J Clin Pathol 115 582 8 11293907 10.1309/84GE-U85A-FMU0-7AUV

[b135-bmi-2007-403] van Imhoff GW Boerma EJ van der Holt B 2006 Prognostic impact of germinal center-associated proteins and chromosomal breakpoints in poor-risk diffuse large B-cell lymphoma J Clin Oncol 24 4135 42 16943530 10.1200/JCO.2006.05.5897

[b136-bmi-2007-403] Villuendas R Pezzella F Gatter K 1997 p21WAF1/CIP1 and MDM2 expression in non-Hodgkin’s lymphoma and their relationship to p53 status: a p53^+^, MDM2-, p21-immunophenotype associated with missense p53 mutations J Pathol 181 51 61 9072003 10.1002/(SICI)1096-9896(199701)181:1<51::AID-PATH689>3.0.CO;2-N

[b137-bmi-2007-403] Visco C Canal F Parolini C 2006 The impact of *P53* and P21(waf1) expression on the survival of patients with the germinal center phenotype of diffuse large B-cell lymphoma Haematologica 91 687 90 16670073

[b138-bmi-2007-403] Vitolo U Botto B Capello D 2002 Point mutations of the BCL-6 gene: clinical and prognostic correlation in B-diffuse large cell lymphoma Leukemia 16 268 75 11840294 10.1038/sj.leu.2402349

[b139-bmi-2007-403] Watanuki-Miyauchi R Kojima Y Tsurumi H 2005 Expression of survivin and of antigen detected by a novel monoclonal antibody, T332, is associated with outcome of diffuse large B-cell lymphoma and its subtypes Pathol Int 55 324 30 15943789 10.1111/j.1440-1827.2005.01832.x

[b140-bmi-2007-403] Went P Dellas T Bourgeau C 2004 Expressionsmuster und prognostische Bedeutung von CD24, *p53* und p21 in Lymphomen, eine Gewebe-Mikroarray-Studie an über 600 Non-Hodgkin-Lymphomen Dtsch Med Wochenschr 129 2094 9 15455300 10.1055/s-2004-831850

[b141-bmi-2007-403] Wilson W Teruya-Feldstein J Fest T 1997 Relationship of *p53*, bcl-2 and tumor proliferation to clinical drug resistance in non-Hodgkin’s lymphomas Blood 89 601 9 9002964

[b142-bmi-2007-403] Winter JN Weller EA Horning SJ 2006 Prognostic significance of Bcl-6 protein expression in DLBCL treated with CHOP or R-CHOP: a prospective correlative study Blood 107 4207 13 16449523 10.1182/blood-2005-10-4222 PMC1895783

[b143-bmi-2007-403] Wlodarska I Veyt E De Paepe P 2005 FOXP1, a gene highly expressed in a subset of diffuse large B-cell lymphoma, is recurrently targeted by genomic aberrations Leukemia 19 1299 305 15944719 10.1038/sj.leu.2403813

[b144-bmi-2007-403] Wright G Tan B Rosenwald A 2003 A gene expression-based method to diagnose clinically distinct subgroups of diffuse large B-cell lymphoma Proc Natl Acad Sci USA 100 9991 6 12900505 10.1073/pnas.1732008100 PMC187912

[b145-bmi-2007-403] Xu Y McKenna R Molberg K 2001 Clinicopathological analysis of CD10^+^ and CD10^−^ diffuse large B-cell lymphoma. Identification of a high-risk subset with coexpression of CD10 and bcl-2 Am J Clin Pathol 116 183 90 11488064 10.1309/J7RN-UXAY-55GX-BUNK

[b146-bmi-2007-403] Yamaguchi M Seto M Okamoto M 2002 De novo CD5^+^ diffuse large B-cell lymphoma: a clinicopathologic study of 109 patients Blood 99 815 21 11806981 10.1182/blood.v99.3.815

[b147-bmi-2007-403] Ye BH Lista F Lo Coco F 1993 Alterations of a zinc finger-encoding gene, BCL-6, in diffuse large-cell lymphoma Science 262 747 50 8235596 10.1126/science.8235596

[b148-bmi-2007-403] Ye BH Chaganti S Chang CC 1995 Chromosomal translocations cause deregulated BCL6 expression by promoter substitution in B-cell lymphoma EMBO J 14 6209 17 8557040 10.1002/j.1460-2075.1995.tb00311.x PMC394745

[b149-bmi-2007-403] Yoshioka T Miura I Kume M 2005 Cytogenetic features of de novo CD5-positive diffuse large B-cell lymphoma, chromosome aberrations affecting 8p21 and 11q13 constitute major subgroups with different overall survival Genes Chromosomes Cancer 42 149 57 15543600 10.1002/gcc.20127

[b150-bmi-2007-403] Zimpfer A Pehrs A Tzankov A 2004 Frequency of CD5-expression and its association with trisomy 3 and 7 in diffuse large B-cell lymphoma. A tissue-microarray analysis of 306 cases Pathol Res Practice 200 288

[b151-bmi-2007-403] Zinzani PL Dirnhofer S Sabattini E 2005 Identification of outcome predictors in diffuse large B-cell lymphoma. Immunohistochemical profiling of homogeneously treated de novo tumors with nodal presentation on tissue micro-arrays Haematologica 90 341 7 15749666

[b152-bmi-2007-403] Zlobec I Steele R Terracciano L 2006 Selecting immunohistochemical cut-off scores for novel biomarkers of progression and survival in colorectal cancer J Clin Pathol10.1136/jcp.2006.044537 PMC2014838 17182662

